# The Relationship between Fluid Milk, Water, and 100% Juice and Health Outcomes among Children and Adolescents

**DOI:** 10.3390/nu14091892

**Published:** 2022-04-30

**Authors:** Elizabeth Gutierrez, Jessica Jarick Metcalfe, Melissa Pflugh Prescott

**Affiliations:** Department of Food Science and Human Nutrition, Division of Nutritional Sciences, University of Illinois Urbana Champaign, Urbana, IL 61801, USA; egutie30@illinois.edu (E.G.); jarick2@illinois.edu (J.J.M.)

**Keywords:** children, milk, juice, water, nutrition, health

## Abstract

Beverages can provide improved nutrient intake and hydration, but also pose concerns related to overnutrition or contamination for children and adolescents who are in a time of critical growth. This narrative review aims to understand the impact of milk, 100% juice, and water consumption on health-related outcomes in youth. The literature review conducted used PubMed, Web of Science, and CABI global. Forty-five research articles met the quality criteria and were included. Health organization and governmental resources were also reviewed to identify current intake and consumption recommendations. All beverages in this review were associated with a variety of desirable and undesirable findings that spanned over 40 different health outcomes. Most studies that assessed milk lacked clear distinction between milk type (flavored vs. unflavored) or fat percentage, making it difficult to understand the impact of milk consumption. The relationship between milk intake and anthropometric-related outcomes were mixed within and across studies. Water was consistently associated with better hydration, while 100% juice and flavored milk intake was associated with more desirable dietary patterns or nutrients that children are currently not consuming adequate amounts of. The implications of these findings were discussed in the context of the National School Lunch Program (NSLP), while considering the impact of issues such as contaminated water and lactose intolerance. This review suggests that water may be an optimal default beverage option in the NSLP to promote hydration and accommodate beverage preferences for those with lactose intolerance.

## 1. Introduction

Globally, more than 340 million children aged 5–19 years old were overweight or obese in 2016 [1]. Studies have shown that diets low in fiber and high in sodium, fat, and refined carbohydrates are related to the development of negative cardiovascular and metabolic outcomes [2] as well as overweight and obesity in children and adolescents [3]. The Dietary Guidelines for Americans (DGA) recognizes diet as a modifiable risk factor for chronic disease [4]. Adequate nutrition is also critical for physical and mental development during childhood and adolescence [5]. The 2020–2025 DGA Scientific Report indicated that vitamin D, potassium, calcium, and dietary fiber are under-consumed, and that the inadequate intake of these nutrients underscores the potential for adverse health outcomes for all Americans [4]. Additionally, the overconsumption of added sugars, saturated fat, and sodium places all Americans at risk for chronic disease [4]. Within adolescents aged 9–14 years old, girls reported inadequate protein consumption, folate, iron, vitamin B6, and vitamin B12 [6,7], while both boys and girls reportedly consumed low amounts of phosphorous, choline, and magnesium [6,7]. Children aged 2–5 years old had an average Healthy Eating Index of 2015 (HEI-15) score of 61 out of 100, while children aged 6–17 years old had a score of 52 out of 100, indicating that young Americans generally do not meet dietary recommendations [8]. A higher total score on the HEI-2015 signifies a diet that better aligns with the recommendations of the DGA. To improve on these areas, the DGA recommends that the consumption of food groups such as whole-grains, vegetables, fruits, and fatty acid ratio be increased, while simultaneously decreasing the intake of sodium, added sugars, and saturated fats within all age categories [4]. While beverages are not a separate category within the HEI, beverages such as fruit juice and milk are disaggregated to the dietary components of interest to better understand dietary patterns surrounding areas like added sugar intake, total fruit, or total dairy [9].

The latest DGA scientific report examines the role of beverages in dietary quality. Beverages were recognized as providing essential nutrients such as calcium, vitamin D, potassium, vitamin C, and magnesium [4]. Yet, beverages were acknowledged for their contributions to added sugars and caloric intake, representing one calorie for every seven calories consumed within children aged 2–19 years old [4]. It is known that sugar-sweetened beverages (SSBs) provide excess energy while often providing few nutritive benefits [10]. Due to the high sugar content, they also raise concern for dental caries. According to the Centers for Disease Control and Prevention (CDC), dental caries is the most common chronic disease in children aged 6–18 years old, despite being highly preventable [11]. Flavored milk, while providing similar essential nutrients to unflavored milk, also contributes excess calories and sugar.

In 2010, the Healthy, Hunger-Free Kids Act (HHFKA) passed, creating improvements to the United States Department of Agriculture’s (USDA) child nutrition programs such as the National School Lunch Program (NSLP), the School Breakfast Program (SBP), and the Summer Food Service Program (SFSP) which included addressing the nutritional quality of the beverages served [12]. To coincide with the DGA, initiatives were made in schools to increase the availability of nutrient-dense foods to improve nutrition and ensure food security to preserve health [13]. Under the HHFKA, beverages within the NSLP and SBP are limited to plain water, low-fat plain milk, fat-free flavored milk, milk alternatives (with an equivalent nutrient profile to milk), and 100% fruit or vegetable juices (with the option to dilute with water) [14]. Free, unlimited potable water is required to be available to children where lunch and breakfast are served [14]. Limitations for the amount of each beverage offered are different for each school level. Beverages such as milk, milk alternatives, 100% fruit, or vegetable juices are limited to 8 ounces for elementary school, with middle school and high school allowing up to 12 ounces for these beverages [14]. There are no ounce restrictions for water [14]. High schools allow for alternative beverage and water options that can be flavored, carbonated, or calorie-free [14]. These options must comply with the Food and Drug Administration’s (FDA) requirement of <5 calories per eight oz beverage, or 10 or fewer calories per 20 fluid oz [14]. Even with HHFKA provisions, there is concern that flavored milk and juice availability may be linked to higher BMI [15,16]. While water does not contribute any excess calories or sugar, contaminated water is another consideration that could negatively affect children (as was seen in Flint, Michigan) [17]. Water contaminated with bacteria or metals is a significant concern due to its severe implications for cognition, the gastrointestinal tract, and overall health [18]. Given these concerns with milk, juice, and water, it is necessary to examine the evidence about the benefits or risks of each beverage to better understand the potential ramifications of their use in child nutrition programs. The aim of this narrative review was to understand the impact of 100% juice, milk, and water consumption on health outcomes, including diet quality, anthropometrics, and cognitive development, within children and adolescents aged 4–18 years.

### 1.1. Nutrient Profile of Fluid Milk

Milk is known to provide critical nutrients that are essential to a balanced diet. These nutrients include calcium, vitamin A, vitamin D (if fortified), phosphorus, riboflavin, vitamin B12, protein, potassium, zinc, choline, selenium, and magnesium [19]. Milk is a significant contributor of calcium in the diet, contributing 22% of daily calcium [20] and over 51% of vitamin D [21] intake to people in the United States (for individuals aged 2 and older). Those who reported milk consumption consumed over 20% of their total daily nutrient intake for vitamin A, vitamin B12, phosphorus, and riboflavin [21]. While milk with higher fat content (2% and whole) contains the same nutrients as milk with lower fat content (fat-free and 1%), low-fat options provide fewer calories per serving [19]. Flavored milk also provides a similar nutrient profile but contributes added sugars and calories [22].

### 1.2. Recommended Intake and Current Consumption Trends of Fluid Milk

The DGA emphasizes a healthy diet that includes fat-free and low-fat (1%) dairy products, with no specific recommendations for whole milk, 2% milk, or flavored milk [23]. They describe a healthy eating pattern as one that includes less than 10% of calories from added sugars and saturated fat [23]. The American Heart Association recommends that children aged 2–18 have less than 25 g of added sugar per day [24]. A half-pint of chocolate milk can have 20–30 g of sugar [25]. The DGA indicates that adding small quantities of sugar might improve the palatability of nutrient-rich foods such as milk, creating the opportunity to consume more nutrient-dense foods [23] (pg. 41). As recommended by the DGA, children aged 5–8 years old should consume the equivalent of around 2½ cups of dairy per day, while three cups are recommended per day for adolescents aged 9–18 years old [23]. There is an age-related decline in milk intake beginning in childhood and continuing into adulthood [21]. Fluid milk accounts for 51% of the total dairy product consumption in Americans, with 75% of fluid milk consumption being attributed to beverages or over cereal [19]. Children aged 4–18 years old are not meeting the recommended intake range for total dairy, with females consuming less than their male counterparts [23]. Looking specifically at milk type, around 20% of children reported consuming fat-free or 1% milk, 45% reported consuming 2% milk, and around 32% reported consuming whole milk [26]. Around 28% of children aged 2–11 reported consuming flavored milk compared to 17% of adolescents aged 12 to 19 [21]. The American College of Gastroenterology recognizes three different forms of lactase deficiency, known commonly as lactose intolerance, that can affect individuals from infancy to adulthood, with around half of American adults living with acquired lactase deficiency that develops with age [27].

### 1.3. Nutrient Profile of 100% Juice

Fruit juice is mainly composed of water with carbohydrates as the second most abundant nutrient [28]. While specific nutrient content varies by fruit used, juices typically contain high amounts of potassium, vitamin A, and vitamin C naturally [28]. After fortification, juice can also provide vitamin D or calcium, in similar amounts to milk, but without the additional nutrients milk provides such as magnesium or protein [28]. Juice has small amounts of protein and other minerals [28].

### 1.4. Recommended Intake and Current Consumption Trends of 100% Juice

The DGA recognizes 100% fruit juice as part of a healthy diet, although excess consumption can lead to excess calories without contributing the same amount of dietary fiber as whole fruits [23]. An American who consumes 2000 calories per day is recommended to have two cups of fruit per day based on the healthy US-Style Eating Pattern [23], which could include 100% fruit juice, as one cup is considered equal to a one-cup serving of whole fruit [23]. The DGA recommends that at least half of fruit intake comes from whole fruit [23]. The American Academy of Pediatrics (AAP) recommends age-appropriate fruit juice servings that contain no added sugars [23], with serving sizes between 4 and 6 ounces per day for children aged 4–6 and 8 ounces per day for children aged 7–18 [29]. Children and adolescents aged 5 to 18 years old were below the recommended daily intake of total fruit, which includes 100% juice, whole fruit, and dried fruit [23]. On a given day, 46.9% of youths aged 2–19 consumed 100% fruit juice as their fruit [30].

### 1.5. Nutrient Profile of Water

Water is crucial for the human body [31] and provides hydration without any contribution to caloric intake [23]. Tap and bottled water can contribute to calcium, magnesium, and sodium intake [32]. Due to water fluoridation, children can acquire the fluoride needed to help prevent cavities [33]. While water contributes beneficial nutrients, lead at any level and manganese at elevated levels are hazardous to children. These minerals at high levels in drinking water have been linked to impacts in IQ level, academic achievement, and attention [34,35,36,37]. Due to lead having no safe level of ingestion, the Flint Water Crisis is an example of how lead is extremely detrimental to developing children and still requires action [37,38]. Similarly, higher manganese concentrations have been shown to be associated with lower cognitive function in children [35,36]. While the vast majority of US drinking water is believed to be safe, there are growing equity concerns about safe water access and skepticism about the rigor of safety monitoring and compliance [39,40]. The Natural Resources Defense Council (NRDC) reports that in 2015 alone there were 18,000 water systems serving 77 million people with violations to the Safe Drinking Water Act, such as exceeding health-based contamination standards, failing to properly test for contaminants, and failing to report contamination [41].

### 1.6. Recommended Intake and Current Consumption Trends of Water

The Institute of Medicine recommends that children, boys, and girls, 4–8 years old, should consume around 7 cups per day of total water [42]. On average, girls aged 9–13 should consume 9 cups of total water per day, while boys should consume 10 cups of total water [42]. Adolescent girls aged 14–18 should consume 10 cups of total water per day, while adolescent boys should consume 14 cups of total water [42]. While the DGA do not have set recommendations for water requirements, they recommend water as a primary beverage choice because it contributes no calories and no added sugars [19]. Total water intake is achieved through drinking water, water in other beverages, and foods containing water [42]. When total water intake from all sources was compared to the Institute of Medicine’s recommendations, it was found that 75% of boys and girls aged 4–8 years old failed to meet the dietary reference intake (DRI), and 83% of girls and 85% of boys aged 9–13 failed to meet the DRI [43]. Tap water consumption was trending upward prior to the Flint Water Crisis, which may have led to increased tap water avoidance in children despite tap water being an affordable option to increase water consumption [44]. Bottled water intake was almost two times more prevalent in children who avoided tap water consumption [44].

## 2. Methods

Three databases (PubMed, Web of Science, and CABI Global) were used to search for peer-reviewed journal articles between July 2020 and July 2021 that had the following search terms: child, adolescents, juice, 100% juice, 100% fruit juice, milk, water, beverage, satiety, weight, obesity, nutrition, consumption, intake, and hydration. A total of 8045 articles were identified in the search of all three databases. The abstracts were reviewed to determine whether they examined the beverages of interest in children and adolescents and met other inclusion/exclusion criteria (see Table 1). Articles that did not provide enough details in the abstract were reviewed in full. We also searched each included article’s reference section for relevant peer-reviewed articles to include. A total of 45 articles met the criteria to be included in this study, of which 31 were cross-sectional, 12 were prospective cohort, 1 was quasi-experimental, and 1 was a non-randomized control trial. The inclusion and exclusion criteria can be found in Table 1. Outcomes of interest included health and diet-related outcomes (e.g., BMI, cholesterol, fruit and vegetable consumption, 24-hour urine osmolality, macronutrient intake). All study designs were included if they met the inclusion and exclusion criteria. The articles were required to examine children between the ages of 4 and 18 years old, as this is the typical age range of NSLP participants [45]. If an article included adults, the results had to include stratification across age groups to allow for the isolation of the results for children in the target age range.

The included articles were assessed for quality using a Quality Criteria Checklist (QCC) that was developed based on the Evidence Analysis Manual (EAL) by the Academy of Nutrition and Dietetics [46]. The QCC developed for this study was based on quality criteria listed in the EAL for each type of study design. The quality criteria assessed items including sampling bias, blinding, intervention duration and intensity, inclusion/exclusion criteria, confounding factors, and the reliability and validity of measures. Two researchers (first and second author) evaluated each article based on the relevant quality criteria. After completing a review of each study, the researchers reviewed the quality criteria scores together, and came to a consensus about any discrepancies between coders. The studies were categorized as strong, moderate, or weak quality based on the composite scoring of the relevant criteria. This included whether studies had specified inclusion or exclusion criteria, involved a representative sample of the population, addressed confounding factors, explained withdrawals, whether measurements of outcomes and risk were blinded, exposure was sufficient to determine the results, and whether the measurements relied on standard, validated instruments. Only studies that were categorized as strong or moderate were included in the final review.

## 3. Results

Of the 45 studies, a total of 12 studies had a quality rating of strong and 33 studies had a moderate quality rating. A summary table of the included studies’ characteristics and outcomes appears below (see Table 2), and outlines study details and quality rating based on the Quality Criteria Checklist. In addition, each study’s results are described to better examine the association between the consumption of each beverage and health and diet-related outcomes. A results matrix (Figure S1: Results Matrix) is included in the Supplementary Materials to visualize the results. 

### 3.1. Research Evidence on the Impact of Milk Consumption

#### 3.1.1. Milk and Macronutrients or Micronutrients

Five studies evaluated the association between milk consumption and the intake of essential macro or micronutrients. Three studies received a strong rating [47,66,69], and two received a moderate rating [56,83]. Study designs included non-randomized control [83], prospective cohort [56,69], and cross-sectional designs [66]. In summary, a positive relationship was found between overall milk consumption and protein intake [56]. No association was found between overall milk consumption and CHO or fat intake [47]. Flavored milk consumption had varying outcomes. Overall, flavored milk consumption had a positive association with CHO [69], fat [69], and calcium intake [47,69,91]. Mixed results were found for saturated fat [66,69], protein [69,83], vitamin D [66,83], dietary fiber [66,69], and potassium [66] intake between studies. Finally, a null association was found when assessing the association between flavored milk consumption and magnesium or sodium intake [66].

#### 3.1.2. Milk and Dietary Patterns or Characteristics

Nine studies examined the relationship between milk consumption and dietary aspects or food consumption patterns. Of the studies, three were rated as strong [47,66,69], and the others were rated as moderate [51,52,56,75,78,83]. The study designs included non-randomized control (*n* = 1) [83], prospective cohort (*n* = 2) [56,69], and cross-sectional designs (*n* = 6) [47,51,52,66,75,78]. Milk consumption (flavored and fat content unspecified) was positively associated with energy intake [47,52,56], fruit, vegetable, and water intake [75], and cereal consumption [51]. Milk consumption (flavored and fat content unspecified) was negatively associated with SSB intake [78]. Flavored milk was positively associated with energy intake [69]. A negative association was found between flavored milk consumption and sugar from non-milk extrinsic sugars, other SSBs, and plain milk [69]. There was an inconsistent relationship between flavored milk and the percentage of kcals from added sugars or saturated fat [66], and added sugar [66,83]. Lastly, a null association was found between flavored milk consumption and diet beverages [69], 100% fruit juice [69], breakfast cereal [69], vegetables [69], and sweets consumption [69].

#### 3.1.3. Milk and Anthropometrics

Nineteen studies evaluated milk consumption and its association with anthropometric outcomes. Six studies received a rating of strong [47,58,64,68,69,90], while the other thirteen studies received a rating of moderate [48,52,53,54,56,57,60,62,65,67,74,79,88]. Study designs included prospective cohort (*n* = 8) [53,54,56,62,64,68,69,79] and cross-sectional designs (*n* = 11) [47,48,52,57,58,60,65,67,74,88,90]. Milk consumption was positively associated with height [53,64] and negatively associated with abdominal obesity [47] and skinfold thickness [56]. Mixed outcomes were observed between milk consumption and weight-for-height [53], weight gain or loss [54], waist circumference [56,60,90], obesity [48,57,58,67,74,79], BMI z-score [53,58,62,65], body fat [56,68], risk of metabolic syndrome [69], fat mass [60,65], and BMI [52,56,60,79,88,90]. Finally, no association was found between milk consumption and waist-to-height [58], weight-for-age [65], waist-to-hip ratio [62], height-for-age [65], waist-to-height ratio [65], or fat-free mass [65].

#### 3.1.4. Milk and Biochemical Indices

Three studies examined the relationship between milk consumption and lab values. All studies received a moderate rating and had a cross-sectional design [50,59,60]. Mixed associations were found between milk intake and 24 h urine osmolality [50,59], HDL [60], LDL [60], and triglycerides [60]. The findings were mixed within the different types of milk consumed (whole and low-fat milk).

#### 3.1.5. Milk and Miscellaneous Outcomes

Three studies examined the relationship between milk consumption and miscellaneous health outcomes. All studies had a moderate rating and were either of prospective cohort [61] or cross-sectional design [60,85]. Overall, a positive relationship was found between milk intake and bone strength [85]. Mixed results were found about the relationship between milk consumption and dental caries [61] and cardiorespiratory fitness [60].

### 3.2. Research Evidence on the Impact of 100% Juice Consumption

#### 3.2.1. 100% Juice and Macronutrients or Micronutrients

Five studies evaluated 100% fruit juice consumption and its relation to child and adolescent macro- or micronutrient intake. The studies received a moderate [70,71,73] or strong rating [72,87]. All studies had cross-sectional designs [70,71,72,73,87]. Overall, 100% fruit juice consumption had a positive relationship with CHO [70,87], vitamin C [70,73], vitamin B6 [70], copper [70], iron [70], and vitamin E intake [73]. Mixed outcomes were found for fiber [70,71,73], fat [70,71,87], SFAs [70,71,87], magnesium [70,73], folate [70,73], potassium [70,73], vitamin A [73], and sodium intake [71]. No association was found between 100% fruit juice intake and protein [87].

#### 3.2.2. 100% Juice and Diet Quality

Two studies evaluated 100% juice intake and its association with diet quality.

The study designs included cross-sectional [71] or prospective cohort [86] and received either a strong [86] or moderate rating [71]. In summary, a positive relationship was found between 100% juice intake and a higher Healthy Eating Index score.

#### 3.2.3. 100% Juice and Dietary Patterns or Characteristics

Five studies evaluated the relationship between 100% fruit juice consumption and dietary patterns. The study designs included prospective cohort [86] or cross-sectional designs [71,75,87,89] and were rated as strong [86,87,89] or moderate [71,75]. In summary, 100% fruit juice consumption was positively associated with energy intake [71,87], fruit intake [71,86,89] whole fruit intake [71,86], water intake [75], unsaturated fat [87], the percentage of energy from CHO [87], and SoFAAs (solid fats, alcoholic beverages, and added sugars) [71]. A negative association was found between 100% fruit juice consumption and the percentage of energy from fat [87]. Mixed associations were found between 100% fruit juice consumption and discretionary fat [71] and total sugar intake [71,87]. No associations were found between 100% fruit juice intake and cholesterol [87], added sugars [87], milk intake [71], or the percentage of energy from protein or added sugars [87].

#### 3.2.4. 100% Juice and Anthropometrics

Eight studies evaluated the association between 100% fruit juice consumption and anthropometrics. The study designs included prospective cohort [63,64] or cross-sectional designs [48,67,70,74,81,87], and were rated as strong [64,87] or moderate [48,63,67,70,74,81]. Between the studies, a mixed association was found between 100% fruit juice consumption and height [64,81], weight [70,81,87], BMI [63,81,87], overweight status [81,87], and obesity status [48,67,74,81,87]. No association was found between 100% fruit juice consumption and waist circumference, skinfold thickness, or body fat percentage [87].

#### 3.2.5. 100% Juice and Biochemical Indices

One cross-sectional study, rated as strong, found that children who consumed 100% orange juice [72] did not have significantly different apolipoprotein, plasma glucose, insulin, and systolic or diastolic blood pressure when compared with non-consumers [72]. Another cross-sectional study rated as moderate found no relationship between 100% juice and 24 h urine osmolality [59].

### 3.3. Research Studies Evaluating the Impact of Water Consumption

#### 3.3.1. Water and Dietary Patterns or Characteristics

Two studies examined whether water intake was associated with dietary patterns. Both studies had a cross-sectional design and a quality rating of moderate. In summary, plain water was not significantly associated with total energy intake within children [80]. Low water consumption was positively associated with less than two drinks of milk per day, less than one drink of non-diet soda, and more than one SSB per day [76]. In addition, low water consumption was also associated with >2 times per day of fruit or 100% fruit juice, three times a day or less of vegetable consumption, and eating fast food one to two times per week or more than three times per week [76].

#### 3.3.2. Water and Anthropometrics

Four studies examined the relationship between water consumption and anthropometric measures. Of the studies, three were rated as strong [16,58,64] and the other had a moderate rating [67]. Study designs included prospective cohort (*n* = 1) [64], quasi-experimental (*n* = 1) [16], and cross-sectional (*n* = 2) [58,67] designs. In summary, water intake was significantly associated with height [64] and negatively associated with BMI and risk of overweight [16]. Water was not found to be associated with obesity [67] or waist-to-height ratio [58].

#### 3.3.3. Water and Biochemical Indices

Four studies evaluated the association between water and hydration status. Of these studies, all four had a cross-sectional design and received a rating of moderate. Three studies found that consumption of water was negatively associated with urine osmolality [49,59,82]. Additionally, a beverage pattern characterized by water and milk had lower 24 h urine osmolality [50]. Overall, children and adolescents who consumed more water had lower urine osmolality [50].

#### 3.3.4. Water and Miscellaneous Outcomes

There were four studies that examined water’s association with physical activity or cognitive function. Of the studies included, one study received a strong rating [58], while the other three studies received a quality rating of moderate [76,77,84]. All studies were of cross-sectional design. To summarize, water was not significantly associated with cognitive function in children aged 10–14 years old [84]. Studies that looked at water consumption and physical activity found differing results. The consumption of water was positively associated with physical activity level [58], while another study found that water intake was not significantly associated with physical activity [77]. Alternatively, low water consumption was negatively associated with being active for at least an hour 5 days per week [76].

## 4. Discussion

All beverages included in this review (milk, 100% fruit juice, and water) were associated with a variety of desirable and undesirable health outcomes. Specifically, milk intake had a positive association with bone strength [85]. This provides an opportunity for osteoporosis prevention due to the essential role of calcium and vitamin D intake during adolescence [91]. Milk consumption was also associated with greater height [53,64], though current research indicates genetics plays the largest role in height attainment [92]. Interestingly, the relationship between milk intake and BMI-related outcomes were mixed within [53,55,60,79] and across studies [52,53,55,56,60,62,65,88]. It is important to highlight that most studies were observational and did not differentiate between flavored and unflavored milk, nor did they differentiate between fat percentage, making it difficult to discern distinct associations across milk types. When milk type was unspecified, there was either a null [56,58,62,65] or inconsistent [55,60] relationship with waist measurements and obesity risk [48,57,58,67,74,79]. Flavored milk consumption was not associated with the consumption of non-milk extrinsic sugar [69], SSBs, added sugars, or the percentage of kcals from added sugars [55]. Both juice and flavored milk were associated with redeeming qualities such as more desirable dietary patterns or the consumption of nutrients that are of concern. While water intake was related to better hydration [49,50,59,82], there were also some findings linking frequent consumers with undesirable dietary patterns [76]. Unlike water, sugar-containing beverages (like flavored milk and juice) nonetheless provide additional calories, highlighting a particular concern when considering the appropriateness of federal subsidies for these beverages within the NSLP and other child nutrition programs.

A cross-sectional study conducted by Fayet et al. found that children who consumed plain milk did not have a significantly different BMI or waist circumference than children who did not consume milk [55]. However, when flavored milk drinkers were added to the study sample, both BMI and waist circumference were significantly higher among milk drinkers than those who did not consume milk, suggesting an undesirable relationship between flavored milk and BMI [55]. A quasi-experimental study carried out by Schwartz et al. was the only other study included in this review that examined the impact of flavored milk on BMI [16]. They concluded that lunchtime water jet access led to a significant decrease in chocolate milk purchases, with no change in white milk purchases (indicating that children who previously consumed chocolate milk switched to water) [16]. A significant decrease in BMI scores was also found [16], suggesting a public health advantage in switching from chocolate milk to water as the default lunch beverage. A recent analysis of school lunch data found that flavored fat-free milk was the leading contributor of added sugars at both school breakfast and lunchtimes [93]. More research is needed to examine the impact of encouraging children to switch from selecting milk with school meals to selecting water, as children generally do not meet calcium and vitamin D recommendations.

The HHFKA requires free, potable water to be accessible during meal service in schools, providing a favorable opportunity as current surveillance approximates that 55% of children are inadequately hydrated [59]. Improving water intake may begin by ensuring that children not only have access to clean, potable water, but also ensuring that children believe the water is safe and clean, as some studies suggest otherwise [94,95]. The Flint Water Crisis remains a current issue that may continue to exacerbate health disparities that are already present in these limited-resource communities [96,97]. Less populated US counties had the majority of their contaminated water violations within areas of lower SES and minority group populations [98]. These areas also had increased odds of initial and repeat water safety violations [98]. Similar disparities exist within urban communities, highlighting that many Americans live with limited plumbing accessibility [99]. Concerns about water safety could explain why calorically dense options such as shelf stable SSBs are more likely to be chosen over water in certain populations [59]. Efforts are needed to improve not only the perception of school water, but also to ensure that HHFKA requirements are being met in all schools. Evidence shows that when water is readily available, children drink water [100]. In addition to mealtime access, schools should promote water consumption by providing “excellent drinking water access in schools”, as previous research has highlighted its importance and need [99]. This study defined excellent drinking water access based on the following criteria: several locations, non-fountain sources, routine maintenance, quality, and safety, yet no school met all criteria in the study [99].

Our findings confirm the relationship between milk and bone strength [101,102] and 100% juice and key nutrients such as potassium, magnesium, and vitamin C [103] found in previous reviews. A review by Feruzzi et al. found similar evidence of low-fat milk drinkers having higher BMIs as this review but concluded that these findings could be related to the parents’ decision [102]. However, our findings suggest that milk’s association with anthropometrics is still hard to discern, and more research is needed to better understand the connection. Feruzzi et al. concluded that, besides the increased risk of tooth decay and minor weight gain in young children and adults, there is no conclusive evidence linking the intake of 100% fruit juice with adverse health effects [102]. Their review included observational, randomized controlled trials, and meta-analyses that included adults, and had studies that used dose–response to assess outcomes.

## 5. Limitations and Strengths

Despite the wide scope of findings in this review, several limitations exist. Most of the studies included were of a cross-sectional design. While epidemiological studies are important, these types of studies make it impossible to isolate the factor that is driving the concluded associations and do not permit causal inference. Future randomized controlled trials would allow researchers to estimate the health impact of children’s consumption of various beverages. Most studies did not stratify their results by flavor or milk fat content, which limits the reader’s ability to understand the true association between the type of milk consumed and health outcomes. An additional limitation was the use of usual intakes to assess who was or was not a consumer of each respective beverage. Of importance, some studies lacked diversity and have limited variability across races and ethnicities, limiting the ability to generalize the findings to a broader population. Although these limitations exist, the current study used the Academy of Nutrition and Dietetics’ Quality Criteria Checklist to exclude weak studies.

## 6. Conclusions and Implications for Practice, Policy, and Research

This review highlights many different benefits and potential drawbacks when considering the position of milk, 100% juice, and water within children’s diets. The widespread adoption of potable water as children’s beverage of choice shows the most promising evidence to ameliorate one of the most pressing global health concerns: obesity. Rates of lactose intolerance in children, especially among African Americans, Native Americans, Jewish individuals, and those with Asian or Hispanic descent [27], further suggest the need to make water the default beverage of child nutrition programs. Canada and some European countries promote water as the primary drink of choice or as the basis of their food guide, yet water is not depicted within MyPlate [104,105]. While the DGA suggest using low-lactose or lactose-free alternatives to achieve recommended intakes, [106] (p. 88), individuals with lactose intolerance require nutritional guidance to ensure that adequate calcium and vitamin D intake are met [107]. School nutrition programs can leverage lower-lactose dairy, such as yogurt or hard cheeses, that may be better tolerated to achieve calcium and vitamin D intake [108,109]. The decrease in fluid milk consumption since 1975 [110] may be leaving room for high caloric beverages that have limited nutrients that are currently of concern, especially for children. Since flavored milk may be preferred over plain milk, our findings suggest that water may be the most appropriate default beverage option in school meal programs given that children may be lactose intolerant and water has no added sugars. Nudge interventions are one approach that has been effective in encouraging children to select healthy options such as water during school mealtimes [111]. 

## Figures and Tables

**Table 1:** Study criteria for inclusion or exclusion.

Inclusion Criteria	Exclusion Criteria
Full-text English	Did not measure milk consumption separately from overall dairy consumption
Published since 2010	Provided specific amounts of beverages for consumption to understand outcomes of varying intake level
Included children between the ages of 4 and 18 years old	Non-100% juice outcomes
Assessed milk, 100% juice, or water consumption	Study did not provide results for the full sample (e.g., only provided results stratified by age group or race)
Focused on health or diet-related outcomes	Intervention studies that aimed to increased beverage consumption through promotional activities (education, reusable water bottles, classes, signs, posters, etc.)

**Table 2:** Summary of included studies characteristics and outcomes.

Authors, Date [Reference #] Study Design Sample Details Quality Rating	Primary Outcome	Results
Abreu et al., 2012 [47] Cross-Sectional Study Age Range: 15–18 *n* = 1209 Portuguese Adolescents Quality Rating: Strong	Examine the influence of milk intake and physical activity on abdominal obesity	↓ Adolescents who had higher milk intake were less likely to have abdominal obesity than adolescents with low milk intake (*p* = 0.006) ↑ Adolescents who had higher milk intake had a higher intake of energy, total calcium, and total protein compared with those who had low milk intake (*p* < 0.05) ⦸ Milk intake was not significantly associated with carbohydrate or total fat intake
Beck et al., 2013 [48] Cross-Sectional Study Age Range: 8–10 *n* = 319 Mexican American Children California Health Interview Survey Quality Rating: Moderate	Determine association between beverage consumption and obesity status in school-aged children	↑ Consumption of 2% milk and water was associated with increased odds of obesity ↓ Consumption of whole milk and flavored milk was associated with lower odds of obesity ⦸ Consumption of skim milk, 1% milk, and 100% fruit juice were not associated with obesity
Bonnet et al., 2012 [49] Cross-Sectional Study Age Range: 9–11 *n* = 529 French Children Quality Rating: Moderate	Measure morning hydration status of children via dietary record and urine osmolality	↓ Water intake (at breakfast) was negatively associated with urine osmolality
Bougatsas et al., 2018 [50] Cross-Sectional Study Age Range: 8–14 *n* = 210 Quality Rating: Moderate	Determine the association between fluid intake patterns and hydration by examining 24 h urine osmolality	↓ Children who had a drinking pattern characterized by water and milk had lower 24 h urine osmolality
Campmans-Kuijpers et al., 2016 [51] Cross-Sectional Study Age Range: 7–18 Dutch Children *n* = 1713 Quality Rating: Moderate	Determine the association between milk consumption and intake of other food products	↑ Milk consumption was positively associated with fruit, vegetable, and cereal consumption
Coppinger et al., 2011 [52] Cross-Sectional Study Age Range: 9–13 *n* = 248 British Schoolchildren Quality Rating: Moderate	Examine the relationship between beverage intake and BMI	↑ Intake of milk and milk-based beverages was associated with total energy intake ⦸ There was no significant association between milk and milk-based beverage intake and BMI (*p* > 0.05)
DeBoer et al., 2015 [53] Prospective Cohort Study Age Range: Birth–5 *n* = 8950 ECLS-B Quality Rating: Moderate	Determine the link between milk consumption and weight and height status in children at age 4 and 5 years old	↑ At age 4, higher milk consumption was associated with greater BMI z-scores, height, and weight-for-height (all *p* < 0.05) ↑ At age 5, higher milk consumption was associated with taller height (*p* < 0.001) ⦸ At age 5, milk consumption was not significantly associated with BMI z-scores or weight-for-height (NS)
Dong et al., 2015 [54] Prospective Cohort Study Age Range: 7–13 *n* = 4646 Quality Rating: Moderate	Assess association between consumption of specific beverages and food and weight gain among children and adolescents	↑ In the change–change model, 3-year excess weight gain was significantly associated with increased intake of full-fat (*p* < 0.01) and low-fat milk (*p* < 0.05) ↓ In the change–level model, higher intake of full-fat and low-fat milk was significantly associated with weight loss (*p* < 0.10)
Fayet et al., 2013 [55] Cross-Sectional Study Age Range: 2–16 *n* = 4487 Australian Children Australian National Children’s Nutrition and Physical Activity Survey Quality Rating: Strong	Evaluate how milk consumption and milk intake patterns influence nutrient intake, meeting of calcium requirements, and anthropometric measures	⦸ At ages 5–8, the BMI of children who consumed exclusively plain milk (16.6 ± 0.2) was not significantly different than children who did not consume milk (16.6 ± 0.2) ↑ At ages 5–8, the BMI of children who consumed both flavored and plain milk (17.1 ± 0.2) was significantly higher than for children who did not consume milk (16.6 ± 0.2) ⦸ At ages 5–8, the waist circumference of children who consumed exclusively plain milk (57.0 ± 0.4) was not significantly different than children who did not consume milk (56.7 ± 0.4) ↑ At ages 5–8, the waist circumference of children who consumed both flavored and plain milk (57.8 ± 0.4) was significantly higher than for children who did not consume milk (56.7 ± 0.4) ⦸ At ages 9–16, the BMI was not significantly different between children who did not consume milk, consumed exclusively plain milk, and consumed both flavored and plain milk ⦸ At ages 9–16, the waist circumference was not significantly different between children who did not consume milk, consumed exclusively plain milk, and consumed both flavored and plain milk
Hasnain et al., 2014 [56] Prospective Cohort Study Age Range: 3–17 (original data at 3–5, followed for 12 years) *n* = 103 Framingham Children’s Study Quality Rating: Moderate	Identify beverage intake patterns’ effect on body fat and composition from childhood into adolescence	↓ Children who had the highest (tertile 3) milk intake in early childhood had less body fat, lower BMI, and lower skinfold thickness in later adolescence than those with the lowest milk intake (tertile 1) ⦸ There was no significant difference between milk intake groups in waist circumference ↑ Children who had the highest (tertile 3) milk intake had higher total energy and protein intake than those with the lowest milk intake (tertile 1) (cross sectional finding at beginning of study)
Hwang et al., 2020 [57] Cross-Sectional Study Age Range: 10–18 *n* = 6121 Korean Children KNHANES Quality Rating: Moderate	Examine association between milk consumption and obesity	⦸ There was no significant association between milk consumption and obesity prevalence
Jomaa et al., 2016 [58] Cross-Sectional Study Age Range: 4–13 *n* = 752 Lebanese Children Quality Rating: Strong	Examine total water intake and the association between water intake and dietary intake in children and adolescents	⦸ Water and milk consumption were not significantly associated with obesity or waist-to-height ratio ↑ Water consumption was higher in children who were physically active than children who were inactive
Kenney et al., 2015 [59] Cross-Sectional Study Age Range: 6–19 *n* = 4134 NHANES Quality Rating: Moderate	Examine whether different beverage intake is associated with urine osmolality	↓ An increase in water intake of 8 oz daily was associated with a significantly lower risk of inadequate hydration (decreased urine osmolality) ⦸ Intake of milk or 100% juice was not significantly associated with hydration status (urine osmolality)
Lahoz-Garcia et al., 2019 [60] Cross-Sectional Study Age Range: 8–11 *n* = 1088 Spanish Schoolchildren Quality Rating: Moderate	Determine the association between dairy intake and adiposity or serum lipid profiles	↓ Consumption of whole milk was negatively related to BMI, waist circumference (WC), fat mass percentage (FM%), fat mass index (FMI), triglycerides, and LDL cholesterol ↑ Consumption of whole fat milk was positively associated with HDL cholesterol and cardiorespiratory fitness (CRF) ⦸ Consumption of whole fat milk was not significantly associated with total cholesterol ↑ Consumption of low-fat milk was positively associated with BMI, WC, FM%, FMI, and triglycerides ↓ Consumption of low-fat milk was negatively associated with HDL cholesterol and CRF ⦸ Consumption of low-fat milk was not significantly associated with total cholesterol and LDL cholesterol
Lempert et al., 2015 [61] Prospective Cohort Study Age Range: 9–15 *n* = 1089 Quality Rating: Moderate	Examine dairy consumption in relation to dental caries experience	↓ High milk intake at age 9 was associated with lower likelihood of having dental carries at age 12 ⦸ Milk intake at age 9 was not significantly associated with the likelihood of having dental caries at age 15
Lin Lin et al., 2012 [62] Prospective Cohort Study Age Range: 11–13 *n* = 3679 Chinese Children (a part of the “Children of 1997” birth cohort) Quality Rating: Moderate	Evaluate the association between dairy product intake and obesity	⦸ Milk consumption at 11 years old was not prospectively associated with BMI z-score or waist-to-hip ratio at age 13
Marshall et al., 2017 [63] Prospective Cohort Study Age Range: 13–17 *n* = 369 from the Iowa Fluoride StudyQuality Rating: Moderate	Assess the association between beverage patterns and anthropometric measures	↓ Participants who were part of the juice cluster had lower average BMIs than participants who were part of the milk or water clusters
Marshall et al., 2018 [64] Prospective Cohort Study Age Range: 2–17 *n* = 717 Quality Rating: Strong	Determine beverage intake’s longitudinal association with nutrient adequacy, energy intake, and height	⦸ There was no significant association between 100% juice intake and height ↑ There was a positive association between milk intake and height ↑ There was a positive association between water intake and height
Nezami et al., 2016 [65] Cross-Sectional Study Age Range: 12–18 *n* = 536 Teen Food and Development Study Quality Rating: Moderate	Examine milk consumption and its association with anthropometric indicators of health	⦸ There was no significant relationship between milk consumption and the following anthropometric measures: BMI z-score, weight-for-age z-score, height-for-age z-score, waist-to-height ratio, fat-free mass, or fat mass
Nicklas et al., 2017 [66] Cross-Sectional Study Age Range: 2–18 *n* = 20,329 NHANES Quality Rating: Strong	Determine flavored milk’s contribution to children’s nutrient intake (calcium, vitamin D, magnesium, fiber, potassium, sodium)	⦸ In children aged 4–18, flavored milk consumption was not associated with fiber, magnesium, added sugars, or sodium ↑ In children aged 4–18, flavored milk consumption was positively associated with intake of vitamin D and calcium ↑ Children aged 4–8 who consumed flavored milk had higher intake of potassium ⦸ In children aged 4–8, flavored milk consumption was not associated with intake of percent of kcals from added sugars, saturated fat, or percent of kcals from saturated fat ↑ Children aged 9–13 who consumed flavored milk had higher intake of potassium and saturated fat (all *p* < 0.001) ⦸ In children aged 9–13, flavored milk consumption was not associated with intake of percent of kcals from added sugars or percent of kcals from saturated fat ↑ Children aged 14–18 who consumed flavored milk had higher intake of percent of kcals from saturated fat (*p* < 0.001) ⦸ In children aged 14–18, flavored milk consumption was not associated with intake of potassium, percent of kcals from added sugars, or saturated fat
Nicklas et al., 2018 [67] Cross-Sectional Study Age Range: 2–18 *n* = 7913 NHANES Quality Rating: Moderate	Determine the association between beverage consumption and weight status	⦸ Consumption of milk was not associated with obesity status ⦸ Consumption of water was not associated with obesity status ⦸ Consumption of 100% juice was not associated with obesity status
Noel et al., 2011 [68] Prospective Cohort Study Age Range: 10–13 *n* = 2245 UK Children Avon Longitudinal Study of Parents and Children Quality Rating: Strong	Determine the association between milk type and weight status in children aged 10–13	↑ Consumption of full-fat milk was associated with lower body fat at age 10 (cross sectional finding at beginning of study) ⦸ Consumption of milk at age 10 (full and reduced fat) was not significantly associated with body fat at age 11 or 13
Noel et al., 2013 [69] Prospective Cohort Study Age Range: 10–13 *n* = 2270 UK children Avon Longitudinal Study of Parents and Children Quality Rating: Strong	Determine the association between flavored milk consumption and dietary intake	↑ Children who consumed flavored milk had higher intake of: kcals, fat, saturated fat, carbohydrates, protein, and calcium ↓ Children who consumed flavored milk had lower intake of: fiber, non-milk extrinsic sugars, sugar-sweetened beverages, plain milk ⦸ Flavored milk consumers and non-consumers did not differ significantly in their consumption of diet beverages, 100% fruit juice, breakfast cereal, fruit, vegetables, or sweets/cookies
O’Neil et al., 2010 [70] Cross-Sectional Study Age Range: 12–18 *n* = 3939 NHANES Quality Rating: Moderate	Determine association between 100% juice consumption and nutrient intake and weight status in adolescents	↑ Children who consumed 100% juice had higher intake of carbohydrates, fiber, vitamin C, vitamin B6, folate, potassium, copper, magnesium, and iron than non-consumers ↓ Children who consumed 100% juice had lower intake of fat and saturated fatty acids than non-consumers ⦸ There were no significant differences between children who consumed 100% juice and non-consumers in terms of weight
O’Neil, Nicklas, Zavonec et al., 2011 [71] Cross-Sectional Study Age Range: 2–18 *n* = 7250 NHANES Quality Rating: Moderate	Determine the difference in diet quality between 100% juice consumers and non-consumers	↑ For children aged 6–18, consumption of 100% juice was positively associated with intake of kcals and fiber ↓ For children aged 6–18, consumption of 100% juice was negatively associated with total sugar intake ⦸ For children aged 6–12, consumption of 100% juice was not significantly associated with intake of total fat, saturated fatty acids, or discretionary fat ↑ For children aged 13–18, consumption of 100% juice was positively associated with intake of total fat, saturated fatty acids, and discretionary fat ↑ For children aged 6–18, consumption of 100% juice was positively associated with total HEI-2005 scores, and intake of fruit (total), whole fruit, and SoFAAS (solid fats, alcoholic beverages, and added sugars) ⦸ For children aged 6–18, consumption of 100% juice was not significantly associated with intake of milk ⦸ For children aged 6–12, consumption of 100% juice was not significantly associated with intake of saturated fatty acids or sodium ↑ For children aged 13–18, consumption of 100% juice was positively associated with intake of saturated fatty acids and sodium
O’Neil, Nicklas, Rampersaud et al., 2011 [72] Cross-Sectional Study Age Range: 2–18 *n* = 7250 NHANES Quality Rating: Strong	Determine the association between 100% orange juice consumption and nutrient intake, diet quality, and other physiological parameters	⦸ Consumers and non-consumers of 100% orange juice did not differ in their systolic blood pressure, diastolic blood pressure, apolipoprotein, plasma glucose, or insulin ↑ Children who consumed 100% orange juice had higher levels of serum vitamin C than non-consumers
O’Neil et al., 2012 [73] Cross-Sectional Study Age Range: 2–18 *n* = 7250 NHANES Quality Rating: Moderate	Determine 100% fruit juice consumption’s association with nutrient intake	↑ Children who consumed 100% fruit juice had significantly higher intake of vitamin C and vitamin E compared to non-consumers ⦸ Children who consumed 100% fruit juice did not differ significantly from non-consumers in fiber intake ⦸ In children aged 6–12, those who consumed 100% fruit juice did not differ significantly from non-consumers in intake of vitamin A, magnesium, folate, or potassium ↑ In children aged 13–18, those who consumed 100% fruit juice had higher intake of vitamin A, magnesium, folate, and potassium
Papandreou et al., 2013 [74] Cross-Sectional Study Age Range: 7–15 *n* = 607 Greek Children Quality Rating: Moderate	Assess beverage intake and its association with overweight/obesity	⦸ There was not a significant association between weight/obesity status and consumption of 100% juice or milk
Park et al., 2011 [75] Cross-Sectional Study Age Range: 12–14 *n* = 4292 Quality Rating: Moderate	Assess the association between low drinking water intake and dietary factors	↑ High intake of 100% juice (drinking three or more times per day) was significantly associated with greater water intake ↑ High intake of milk (two or more glasses of milk per day) was significantly associated with greater water intake
Park et al., 2012 [76] Cross-Sectional Study Age Range: 9–12 *n* = 11,049 National Youth Physical Activity and Nutrition Study Quality Rating: Moderate	Examine whether low water intake is associated with other less favorable dietary and behavioral factors	↑ Low water consumption was positively associated with <2 glasses per day of milk, less than one drink of non-diet soda, more than one sugar sweetened beverage per day, >2 times per day for fruit or 100% fruit juice, eating vegetables less than three times per day, eating fast food once or twice per week and more than three times per week, and being active for at least an hour 5 days per week
Perales-García et al., 2018 [77] Cross-Sectional Study Age Range: 7–12 *n* = 242 Spanish Children Quality Rating: Moderate	Evaluate dietary water intake and hydration status in school-aged children to determine whether there is an association with PA/sedentary behavior	⦸ Water intake was not significantly related to physical activity behaviors
Rangan et al., 2012 [78] Cross-Sectional Study Age Range: 8–10 *n* = 222 Australian Children Quality Rating: Moderate	Determine the association between dairy intake and diet quality	↓ Consumption of milk was negatively associated with consumption of sugar-sweetened beverages
Scharf et al., 2013 [79] Prospective Cohort Study Age Range: 2–4 *n* = 10,700 ECLS-B Quality Rating: Moderate	Determine the association between type of milk consumed and BMI z-score/overweight/obese status in preschool-aged children	↑ At age 4, overweight/obese children consumed more 1% and skim milk than healthy weight children (*p* < 0.01) (cross-sectional finding) ↑ At age 4, BMI z-scores were lower for children who consumed 2% and whole milk than children who consumed 1% and skim milk (*p* < 0.01) ↑ At age 4, linear regressions showed that consumption of higher fat milk was associated with lower BMI z-scores (*p* < 0.001)
Schwartz et al., 2016 [16] Quasi-Experimental Study Age Range: New York Elementary- and Middle School-Aged Children *n*= 1,065,562 Quality Rating: Strong	Examine the effect of a water jets initiative on BMI, overweight, and obesity	↓ Adoption of water jets was associated with a reduction in BMI z-scores ↓ Adoption of water jets was associated with a reduction in likelihood of being overweight
Shamah-Levy et al., 2016 [80] Cross-Sectional Study Age Range: 5–11 *n* = 2536 Mexican Children Quality Rating: Moderate	Evaluate the association between plain water intake and total energy intake in Mexican school-aged children	⦸ Plain water consumption was not significantly associated with total energy intake
Shefferly et al., 2016 [81] Cross-Sectional Study Age Range: 2–5 *n* = 8950 ECLS-B Quality Rating: Moderate	Examine the relationship between 100% fruit juice consumption and changes in early childhood height, weight, and BMI	↑ Children who drank 100% juice consistently at age 2 had greater increases than non-drinkers in BMI z-score and weight z-score by age 4 ↑ Children who drank 100% juice consistently at age 2 had higher odds of becoming overweight by age 4 than non-drinkers ↓ Children who drank 100% juice consistently at age 2 had smaller increases than non-drinkers in height z-score by age 4
Stookey et al., 2012 [82] Cross-Sectional Study Age Range: 9–11 *n* = 548 Quality Rating: Moderate	Evaluate cell hydration status by assessing dietary records and urine osmolality	↓ Drinking water was inversely associated with urine osmolality
Thompson et al., 2020 [83] Non-Randomized Controlled Trial Study Age Range: Middle- and High School-Aged Children and Adolescents *n* = 24 schools, ~3062 Quality Rating: Moderate	Assess the effect of a chocolate milk removal policy on selection, consumption, and waste to determine nutrient intake	⦸ Changes in calcium, protein and vitamin D intake were not significant after chocolate milk was removed from cafeteria ↓ Consumption of added sugar from milk declined significantly after chocolate milk was removed from cafeteria
Tung et al., 2020 [84] Cross-Sectional Study Age Range: 10–14 *n* = 230 Malaysian Children Quality Rating: Moderate	Examine the association between fluid intake, hydration, and cognitive function	⦸ Water intake was not significantly related to cognitive function
Uenishi and Nakamura, 2010 [85] Cross-Sectional Study Age Range: 15–18 *n*= 38,719 Japanese Adolescents Quality Rating: Moderate	Determine the association between dairy product intake and bone strength	↑ Milk intake was significantly associated with osteo-sono assessment index (bone strength)
Wan et al., 2020 [86] Prospective Cohort Study Age Range: 3–16 *n* = 100 Quality Rating: Strong	Examine association between consumption of 100% fruit juice during preschool and subsequent diet quality and change in BMI throughout childhood	↑ Consumption of 100% fruit juice during preschool years was associated with higher HEI 2015 scores, and consuming more fruit (total) and whole fruit during adolescence ⦸ There was no significant association between 100% fruit juice consumption during preschool years and BMI in adolescence
Wang et al., 2012 [87] Cross-Sectional Study Age Range: 4–18 *n* = 5856 NHANES Quality Rating: Strong	Determine the association between 100% orange juice consumption and macronutrient intake, energy intake, and body composition	↑ Consumption of 100% orange juice was positively associated with intake of kJs, kcals, carbohydrates, total sugar, total fat, SFAs, MUFAs, PUFAs, and % energy from carbohydrates ↓ Consumption of 100% orange juice was negatively associated with % energy from fat ⦸ Consumption of 100% orange juice was not significantly associated with intake of protein, added sugars, cholesterol, % energy from protein, or % energy from added sugars ⦸ Consumption of 100% orange juice was not significantly associated with weight-for-age z-score, BMI, waist circumference, skinfold thickness, body fat %, or overweight/obesity status
Wiley 2010 [88] Cross-Sectional Study Age Range: 2–10 *n* = 2526 NHANES Quality Rating: Moderate	Examine the association between milk consumption and BMI	⦸ For children aged 5–10, milk consumption was not significantly associated with BMI
Yang et al., 2013 [89] Cross-Sectional Study Age Range: 4–adult *n* = 12,971 NHANES Quality Rating: Strong	Evaluate impact of 100% orange juice consumption on the diet	↑ Consumption of 100% orange juice was positively associated with consumption of fruit (from whole fruit and fruit juices)
Yuzbashian et al., 2021 [90] Prospective Cohort Study Age Range: 6–18 *n* = 531 Quality Rating: Strong	Examine the association between total and individual dairy food consumption and incidence of metabolic syndrome in children and adolescents	↓ Higher consumption of low-fat milk was associated with a lower risk of metabolic syndrome ⦸ Consumption of high-fat milk was not significantly associated with risk of metabolic syndrome

## Data Availability

Not applicable.

## Supplementary Materials

The following are available online at https://www.mdpi.com/article/10.3390/nu14091892/s1, Figure S1: Results matrix.
