# Supplementary material for: The Relationship between Fluid Milk, Water, and 100% Juice and Health Outcomes among Children and Adolescents
Source: Nutrients. 2022 Apr 30;14(9):1892. doi: 10.3390/nu14091892 (PMC9100844; doi:10.3390/nu14091892)
Supplement: Supplementary file 1 [file nutrients-14-01892-s001.zip › nutrients-1683148-supplementary.pdf]

Supplementary Figure S1. Results Matrix

This figure provides the desirable (green), undesirable (red), mixed (yellow), or null (grey) findings across the studies included in this literature review. Outcomes are categorized into five overarching groups: macronutrients and micronutrients, dietary aspects and patterns, anthropometrics, biochemical indices, and miscellaneous.

**Outcome Category**	
**Macronutrients and Micronutrients□**	
**Carbohydrates** (includes % energy from carbohydrates)	70 48 88 71
**Fat** (Total Fat, Fat intake, % energy from fat)	48 70 88 74 71
**Saturated Fat** (includes % energy from sat. fat)	67 70 88 71 74
**Unsaturated Fat** (Monounsaturated and polyunsaturated)	88
**Protein** (includes % energy from protein)	57 70 84 48 88
**Dietary Fiber**	67 70 73 74 71

**Fat-Soluble Vitamins** (Vitamin A, E, D)	67 84 74
**Water-Soluble Vitamins** (Folate, Vitamin C, B6)	71 74
**Minerals** (Calcium, magnesium, manganese, potassium, sodium, iron, copper)	70 48 84 67 74 71
**Dietary Aspects & Patterns**	
**Energy Intake*** (Includes energy intake, Kcal, KJ intake)	84 67 57 76 16 87 74
**Sugar Intake** (non-milk extrinsic sugars, total sugar, added sugar, % energy from added sugars)	84 70 67 88 72
**Discretionary Fats** (Discretionary fat, cholesterol, SOFAAs)	72 88

**Fruit Intake** (Includes whole fruit)	52 70 77 72 90 87
**Vegetable Intake**	52 70 77
**100% Juice Intake**	70 77
**Milk Consumption** (Includes milk intake, plain milk intake)	70 77 72
**Water Intake**	76 76
**SSB Intake**	70 79 77
**Diet Beverages**	70 77
**Sweets/Cookies/Fast Food**	70 77

**Cereal Intake**	52 70
**Diet Quality** (Includes HEI 2005 and 2015)	72 87
**Anthropometrics**	
**Height-related×** (Includes height, height for age z-score, height z-score)	54 65 66 65 65 82
**Weight-related** (Includes body weight, weight for age z-score, weight for height z-score, weight z-score, weight gain)	66 54 55 71 88 82
**BMI Related Outcomes** (Includes BMI z-score, BMI percentile)	57 61 54 56 80 66 63 89 53 16 82 88 64
**Overweight/Risk of Overweight+**	16 88 82
**Obesity/Risk of Obesity+**	

	75 80 49 58 68 59 59 49 68 68 75 88 49
**Body Fat** (Includes fat mass, body fat, body fat %, skinfold thickness, abdominal obesity)	57 69 66 61 48 88
**Fat-Free Mass**	66
**Waist Measurements∞** (Includes waist to height ratio z-score, waist circumference, waist to hip ratio*)*	56 61 59 66 57 63 59 88
**Risk of Metabolic Syndrome**	91
**Biochemical Indices**	
**24-hour Urine Osmolality**	60 51 51 83 60 50 60
**Blood Pressure**	73

**Apolipoprotein**	73
**Blood Glucose**	73
**Insulin Levels**	73
**Serum Vitamin C**	73
**Total Cholesterol**	61
**HDL**	61
**LDL**	61
**Triglycerides**	61
**Miscellaneous**	
**Cognitive Function**	85

**Physical Activity**	59 77 78
**Dental Caries**	62
**Bone Strength**	86
**Cardiorespiratory Fitness**	61

Footnotes:

□*In this table, we classified higher nutrient density of any macro- or micro-nutrients as desirable.*

**In this table, we classified higher energy intake as an undesirable outcome.*

×*In this table, we classified greater height as a desirable outcome.*

+*In this table, we classified overweight, or obesity has an undesirable outcome.*

∞*In this table, we classified a higher waist circumference as an undesirable outcome.*

~*This study looked at low water intake.*
